# Relevance of the Iron Distribution in Natural Smectite Clays for the Thermal Stability of PMMA–Clay Nanocomposites

**DOI:** 10.1021/acsomega.4c04751

**Published:** 2024-08-16

**Authors:** Camila R. Ferreira, Celso V. Santilli, Valérie Briois, Sandra H. Pulcinelli

**Affiliations:** † Chemistry Institute, São Paulo State University, Araraquara, São Paulo 14800060, Brazil; ‡ Synchrotron SOLEIL, UR1-CNRS-SOLEIL, L’Orme des Merisiers, Saint-Aubin 91192, France

## Abstract

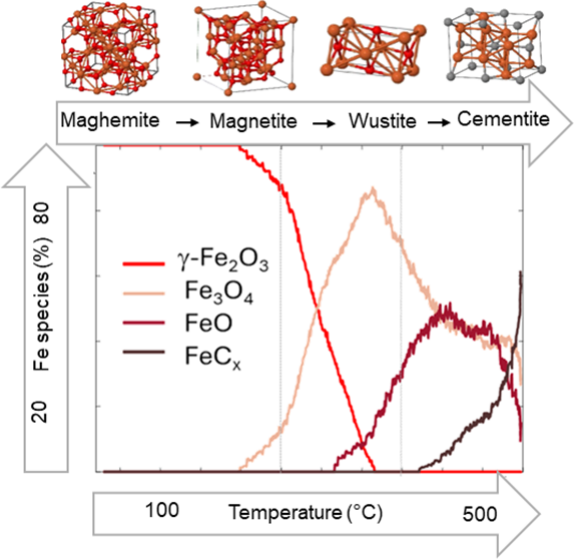

Polymer–clay nanocomposites have greater thermal stability compared to the pristine polymer matrix. This can be attributed to the physical barrier provided by the inclusion of 2D clay nanoparticles (especially of the smectite group), together with radical trapping related to the distribution of specific 3d atoms in the inorganic phase. To elucidate the relevance of the Fe^3+^ distribution in this synergic effect, the iron atoms present in octahedral sheets of natural nontronite clay (Non, 5.6 wt % Fe) or in maghemite (M) nanoparticles (γ-Fe_2_O_3_) were incorporated in a poly(methyl methacrylate) (PMMA) matrix. Na-laponite (Lap) clay was used to evaluate the contribution of the diffusion barrier effect to the increased thermal stability of a PMMA-Lap nanocomposite, as evidenced by the upshift of the thermogravimetric (TGA) curve compared to that for PMMA. The contribution of radical trapping to the thermal stability of the PMMA-Non nanocomposite was evidenced by a significant shift of the Fe K-edge rising edge position by −4.5 eV after iron reduction by heating in N_2_, while similar treatment of pristine nontronite did not lead to a significant rising edge shift in the X-ray absorption spectra (XAS). This downshift demonstrated the reduction of Fe^3+^ to Fe^0^, induced by the sequestration of radicals formed by PMMA depolymerization. Raman spectroscopy analysis evidenced the formation of graphitic char deposits above 400 °C, further improving the thermal stability of PMMA-Non by providing an additional physical barrier to mass transport. A fourth contribution of well-dispersed iron was the abstraction of carbon from the char by the iron carburization reaction, which hindered CO_2_ formation by oxidative coking. In contrast, no relevant contribution of graphitic layer deposition was observed for the PMMA-M-Lap nanocomposite, where its improved thermal stability was only due to the combined contributions of the gas diffusion barrier effect and radical trapping by iron atoms. The maghemite effectively captured the radicals confined by the clay sheets, resulting in significant stabilization of the nanocomposite, with a shift of the mass loss of the PMMA-M-Lap nanocomposite compared to PMMA-Lap.

## 1 Introduction

Highly flammable polymers such as poly(vinyl chloride), polystyrene, and poly(methyl methacrylate) (PMMA) are used in construction materials including pipes and fittings, insulation, and window and door glasses, which in the event of a fire produce heat, smoke, and toxic gases.^1−4^ Examples of devastating fire with worldwide impact occurred in France in 1970 and in Brazil in 2013, resulting in the loss of about 146 and 231 lives, respectively. The predominant cause of deaths was the release of toxic gas and smoke generated by the ignition of polyurethane (PU) and other polymers present in the ceiling, walls, and seats.^5,6^

The thermal properties of polymeric organic materials can be enhanced through the incorporation of inorganic particles.^7−9^ During the last 30 years, conventional micrometer-sized inorganic particles have been progressively replaced by nanometric ones, for the preparation of so-called nanocomposites with new properties conferred by the very large increase of the specific area of the interface between the particles and the polymeric matrix.^10^ Among nanoscale inorganic fillers, layered clays have been used to prepare nanocomposites, offering outstanding performance, which would not be achieved using conventional composites.^11^ The wide interest in polymer–clay nanocomposites (PCN) reflects the easy availability, environmental attributes, and low cost of clay minerals, with only a small weight percentage of nanoclay being required to produce useful materials.^12^

The flame retardance mechanism typically involves the formation of a high-performance carbonaceous-silicate char, which accumulates at the polymer/clay interface during combustion. This results from a change in the polymer degradation pathway in the presence of clay particles with lamellar morphology, which act as physical obstacles in the diffusion paths of the permeating molecules, creating a tortuosity effect. Formation of the carbonaceous-silicate char insulates the underlying material and decreases the rate of polymer mass loss.^13−16^ However, in the production of PCN, the incompatibility between the two materials must be considered since layered clays are predominantly hydrophilic in nature, while the organic polymer is predominantly hydrophobic. In this system, the nanometric dispersion of the layered clay into a polymer matrix can be achieved by modification of the clay surface with organic groups, involving ionic exchange with cations or anions present in the interlayer space or grafting reactions of hydrophobic groups.^17^ Grafting is usually performed by reactions between silane coupling agents and reactive silanol groups located at the broken edges of the clay platelets and at the structural defects located at the interlayers and the external surface.^18,19^

Other emerging inorganic additives with promising effects on polymer thermal degradation are transition metal oxide nanoparticles.^20−24^ For instance, the incorporation of Fe_2_O_3_ or TiO_2_ nanoparticles in a PMMA matrix improves the thermal stability of the polymer due to the reduction of radical mobility by restriction of the segmental motion of the chains, as a result of steric hindrance.^22,23^ Recent reviews have reported better thermal performance of PCN containing metal oxide/hydroxide (TiO_2_/Al(OH)_3_), attributed to improvement of the exfoliating characteristic of the organo-modified clay nanoparticles.^25−28^ However, as pointed out previously, the polymer decomposition mechanism is affected by the physical and chemical features of clays.^29,30^ In the case of PMMA–clay nanocomposites prepared with natural montmorillonite containing different amounts of Fe^3+^ in the octahedral sheets, the reversible reduction to Fe^2+^ induced by chemical reactions with the radicals formed during PMMA decomposition was shown to be responsible for increased polymer thermal stability.^29^ A greater amount of Fe^3+^ in the octahedral montmorillonite sheets was associated with a better thermal stability of the nanocomposite. A study of a PMMA–synthetic clay nanocomposite with reduced thermostability found that the radical scavenging of this polymer^31^ by 3d cations was only effective when the layered edifice was preserved during heating.^30^ Under such conditions, the nanoconfined environment provided by the clay layers, which restricted the movement of polymer chains, hindered the propagation of radicals and increased their extinction by reduction of the 3d cations still present in the octahedral sheets.

Several mechanisms proposed to explain the improved thermal stability of PCN are based on the role of the high aspect ratio of the clay lamellae, which could contribute to the diffusion barrier and heat transfer effects, preventing the entry of oxygen and the escape of volatile components.^12,30,32^ In addition, the nanoconfinement created by the clay layers could favor radical recombination, consequently delaying depolymerization.^33^ The presence of transition metals such as Fe, Cu, Zn, and Ti in clays can increase the thermal stability of polymers, with several interpretations having been proposed for the role played by these metals.^31,32,34,35^ The presence of iron in clays favors the so-called radical trapping mechanism,^36^ but there is a lack of consensus in the literature about which mechanism is predominant since other possible mechanisms can involve the clay lamellae diffusion barrier, the char layer diffusion barrier, radical recombination, and gaseous diluents. In addition, the mechanism related to the presence of iron oxide nanofillers in PMMA, either alone or in combination with clays, is not well-known, as shown by the results reported in the literature.^25,29,33,37^

In this work, nanocomposites consisting of PMMA as a polymer matrix, together with clays (with and without Fe) and iron oxide nanofillers, were prepared by using in situ MMA radical polymerization and the inclusion of covalently functionalized smectite clay or maghemite nanoparticles. The findings assist in elucidating the contributions of the different mechanisms suggested to explain the greater thermal stability of PMMA–clay nanocomposites containing iron. The development of the PCN and understanding the phenomena occurring during thermal degradation of the polymer required multimodal characterizations employing different physicochemical analysis techniques. To this end, in situ X-ray absorption spectroscopy (XAS) coupled with Raman spectroscopy and mass spectrometry measurements were carried out to understand the thermal degradation of PMMA–clay nanocomposites by correlating thermogravimetric events (identified using TGA) with the thermal evolution of the electronic structure and the local order environment of the iron transition metal (determined using XAS).

Hence, the focus of the present work was to determine the influence of Fe ions located in the clay, demonstrating the reduction of Fe^3+^ ([Ar]3d^5^]) to Fe^0^ ([Ar]3d^6^4s^2^), as well as to identify the iron species formed during thermal decomposition of the PMMA–clay nanocomposite. PMMA–nontronite (PMMA-Non) nanocomposites were used to determine the influence of iron ions present in the octahedral sheet lamellae domains on the scavenging of radicals, in comparison with pristine maghemite (M) and PMMA–maghemite (PMMA-M), where iron was present only in the γ-Fe_2_O_3_ lattice structure, as well as with PMMA–maghemite–laponite (PMMA-M-LaP), where γ-Fe_2_O_3_ was added to Fe-free clay. The purpose of selecting this set of samples was to isolate the physical barrier contribution from the radical trapping contribution in the thermal degradation mechanisms of PCN, presenting different spatial distributions of Fe^3+^.

## 2 Experimental Section

### 2.1 Reagents and Fillers

All the reagents and fillers were commercially available as follows: standard Fe-free laponite (LNa-RD, BYK Additives and Instruments), Fe-bearing nontronite (NG-1, Source Clays Repository of the Clay Minerals Society, USA), maghemite iron oxide (γ-Fe_2_O_3_, 99%, 20–40 nm, US Research Nanomaterials, Inc.), methyl methacrylate (MMA, 99% purity, containing ≤30 ppm hydroquinone inhibitor, Fluka), 3-(trimethoxysilylpropyl) methacrylate (TMSM, 98% purity, Fluka), tetrahydrofuran (THF, anhydrous, >99% purity, no inhibitor, Sigma-Aldrich), and benzoyl peroxide (BPO, Sigma-Aldrich). The chemicals (except MMA) were used as received. MMA was distilled to remove the polymerization inhibitor and impurities and was stored in a freezer prior to use.

Modification of the laponite (Lap) and nontronite (Non) clays with the TMSM coupling agent was performed according to an adaptation of the procedure described by Shanmugharaj et al.^38^ for grafting of aminopropyltriethoxysilane. A 1 g portion of the clay, previously dried at 60 °C for 24 h, was transferred to 100 mL of THF with magnetic stirring until complete dispersion was achieved. Separately, 1 g of TMSM was dissolved in 100 mL of THF. This solution was added to the clay mineral dispersion, which was then stirred for 12 h. The solid was separated and dried at 60 °C for 24 h. A similar procedure was used to functionalize the maghemite (M) nanoparticles.

To prepare the γ-Fe_2_O_3_–laponite (M-Lap) sample, maghemite (0.1 M aqueous dispersion) was added to 1 g of delaminated laponite clay (from dispersion in 100 mL of deionized water under agitation for 24 h) and kept under agitation for 24 h at 70 °C. The resulting sample was dried at 100 °C for 3 h.

### 2.2 Preparation of Nanocomposites

The nanocomposites were prepared by in situ MMA radical polymerization in a dispersion of the functionalized clay (or iron oxide) in THF. The functionalized clay was dispersed in a flask containing 25 mL of THF, with 10 kHz ultrasonication for 3 h, followed by the addition of 0.01 M MMA and keeping the dispersion under magnetic stirring for 24 h. The benzoyl peroxide initiator was added (BPO/MMA molar ratio of 0.01), and the polymerization reaction was performed under magnetic stirring at 70 °C for 15 h. The sample was poured into a polytetrafluoroethylene vessel, dried at room temperature (RT) for 24 h, and finally dried at 100 °C for 3 h. The resulting nanocomposites contained 15% (w/w) of clay–TMSM, nearly 11% of which was clay, and 15% of maghemite. Elemental analysis of the different clays used for preparing the nanocomposites was performed by ICP-OES (inductively coupled plasma–optical emission spectroscopy), using a PlasmaQuant 9000 instrument (Analytik Jena). The results are shown in Table S1, together with the sample nomenclature employed.

### 2.3 Characterizations

Thermogravimetric analyses (TGA) of 10 mg portions of the nanocomposite powders were performed using an SDT Q600 system (TA Instruments) under a 100 mL min^–1^ flow of N_2_ with heating at a rate of 10 °C min^–1^.

Fe K-edge X-ray absorption spectra of the nanocomposites and the pristine clays were recorded in situ during heating from RT to 550 °C (at 10 °C min^–1^), maintaining the final temperature for 30 min, using the Quick-EXAFS ROCK beamline of the SOLEIL synchrotron radiation facility.^39^ The time for the acquisition of each Quick-EXAFS spectrum was 0.25 s. At the end of the thermal treatment, the sample was cooled, and data were collected at RT. The time-resolved data set enabled the determination of the effect of radical trapping on polymer thermal stability caused by the iron atoms present in the clay. The Quick-EXAFS measurements were carried out in situ, with simultaneous online analysis of gaseous thermal decomposition products by mass spectrometry (Cirrus LM99 analyzer, MKS), as well as Raman spectroscopy analysis of the solid samples (RXN1 analyzer, Kaiser Optical Systems, Inc.), where the beam of a solid-state diode laser operating at 532 nm was focused on the bulk region of the sample, using a 150 mm working distance objective lens with 10× magnification. The Raman spectroscopy technique, which is very sensitive to the polymeric matrix, can provide insights regarding the polymer degradation temperature and the resulting char formation and is helpful for correlating results obtained by XAS.

The iron-based chemical species involved in the thermal decomposition of the nanocomposites were identified by a multivariate analysis of the in situ XAS data. The principle of the multivariate curve regression with alternating least-squares (MCR-ALS) fitting procedure was based on decomposition of the matrix data set D(*ij*) containing *i* XAS spectra of the sample, recorded at time *t*_i_, for *j* energy values, according to the relation
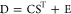
1where E is a matrix containing the residual noise^40−42^ and C(*in*) and S^T^(*nj*) are matrices containing the concentration profiles and XAS spectra of the *n* pure components involved during the thermal decomposition of the sample, respectively.^40−42^ Prior to starting the MCR-ALS minimization, principal component analysis with singular value decomposition (PCA-SVD)^43,44^ was carried out to determine the number of pure components for each sample.

## 3 Results and Discussion

### 3.1 Thermal Stabilization of the Nanocomposites

**Figure 1:**
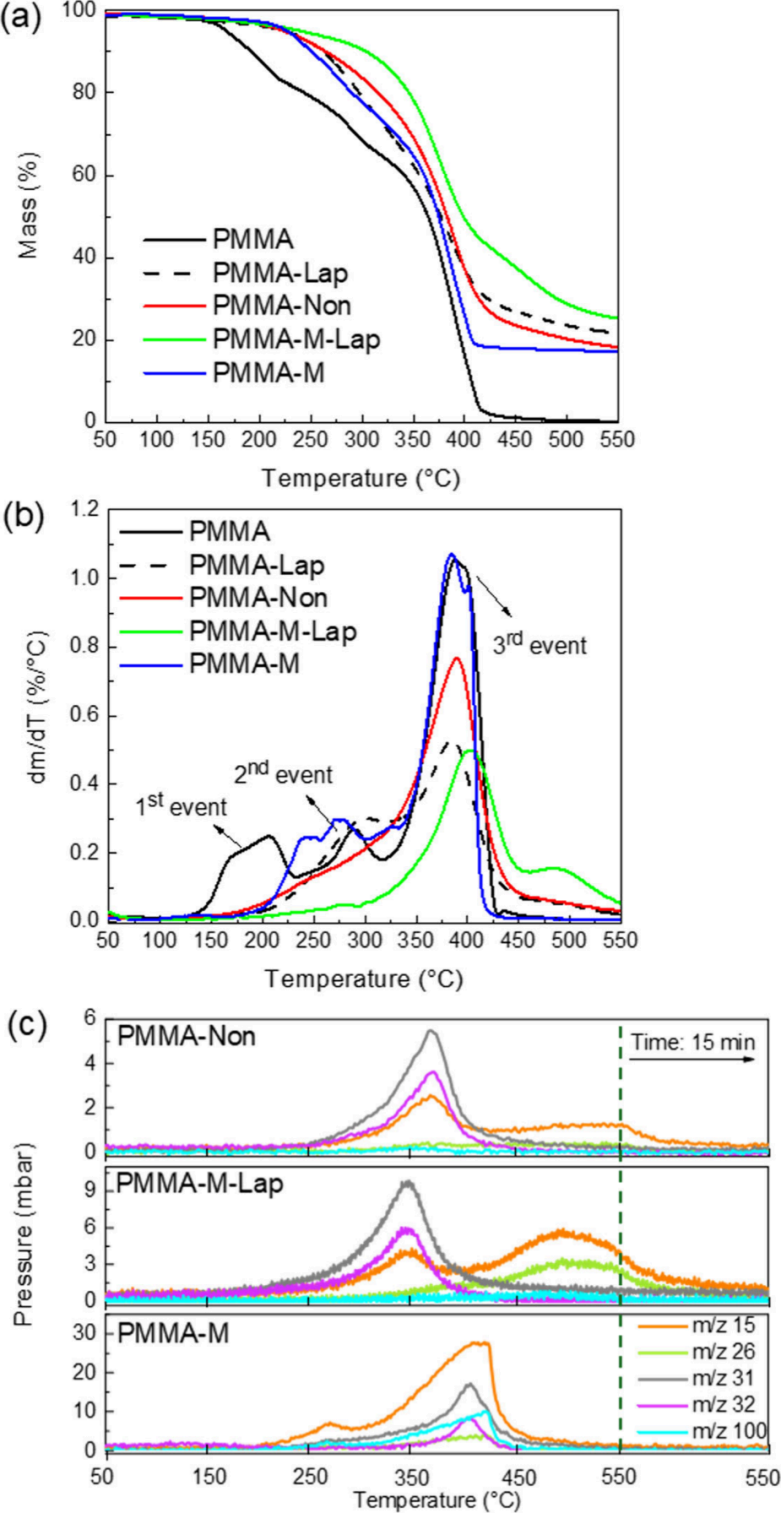
Comparison of the thermogravimetric behaviors of the PMMA-Non, PMMA-Lap, PMMA-M, and PMMA-M-Lap nanocomposites and pristine PMMA: (a) TGA and (b) dTGA curves. (c) Evolution of the products of decomposition monitored by mass spectrometry for the PMMA-Non, PMMA-M-Lap, and PMMA-M nanocomposites.

The effects of clay incorporation and iron dispersion on the thermal stabilization of PMMA were evaluated by comparison of the thermogravimetric curves (Figure 1a) corresponding to the PMMA-Non, PMMA-Lap, PMMA-M, and PMMA-M-Lap nanocomposites with that for pristine PMMA. As reported previously,^45^ thermal decomposition of pristine PMMA occurs in three steps, as clearly evidenced from the first derivative (dTGA) of the TGA curve (Figure 1b(b)), attributed to a depolymerization sequence involving head-to-head linkages, vinylidene end groups, and random unzipping. The thermal stabilization of PMMA in the nanocomposites was evidenced by the substantial upshift of the onset of thermal degradation, due to the almost complete suppression of the lower temperature step (*T* < 230 °C). For example, if the temperature for 10% mass loss (*T*_10%_) is considered, there was an increase of 112 °C for the PMMA-M-Lap nanocomposite compared to PMMA. The mass loss profiles for PMMA-M and PMMA-Lap were almost identical up to 370 °C, showing that the physical barrier or radical trapping mechanisms were equally effective in delaying degradation when they acted alone. Above this temperature, the rate of mass loss was higher for PMMA-M than for PMMA-Lap, evidencing that the additional contribution of the physical barrier provided by the clay favored the trapping of thermal decomposition products, which reduced the total mass loss of the PCN. This effect was even more evident for the PMMA-M-Lap nanocomposite, where greater thermal stabilization, characterized by a deceleration of the mass loss from 400 °C, indicated the synergic contributions of the clay layered edifice and the iron oxide nanoparticles.

The gases produced in each thermal decomposition step of the PMMA-Non, PMMA-M-Lap, and PMMA-M nanocomposites were analyzed by mass spectrometry in parallel with EXAFS and Raman analyses. As shown in Figure 1c, the partial pressures of different products of PMMA decomposition increased above 250 °C, highlighting the stepped profile of the monomer fragment (MMA, *m*/*z* = 100). This fragment was one of the dominant products of PMMA decomposition for the PMMA-M sample. As expected for free radical depolymerization (or polymerization),^46^ the side reactions blocking MMA formation produced methyl (CH_3_, *m*/*z* = 15) and methoxyl (OCH_3_, *m*/*z* = 31) radicals, which could then abstract a hydrogen from another species to form methanol (CH_3_OH, *m*/*z* = 32), ethylene (C_2_H_4_, *m*/*z* = 26), carbon dioxide (CO_2_, *m*/*z* = 44), and char. The release of methanol, methyl, and methoxyl dominated the thermal decomposition products of the PMMA-Non and PMMA-M-Lap samples. The almost complete absence of MMA for these clay-containing nanocomposites (Figure 1c) highlighted the importance of the barrier effect provided by clay minerals. The broad double partial pressure maximum was in good agreement with the events observed in the dTGA curves, reflecting the complexity of the PMMA degradation. The degradation mechanism consisted of the following sequence of reactions: (i) initiation with random scission of head–tail linkages, resulting in the formation of end-chain radicals; (ii) propagation, related to β-scission of active centers, forming smaller unsaturated species such as unsaturated PMMA, liable to further degradation; and (iii) termination, which could occur by recombination and disproportionation of radicals, ending the degradation cycle. The roles of the iron distribution and the confinement caused by the clay on this stepped thermal PMMA decomposition mechanism are discussed in the next sections.

### 3.2 Relevance of Iron Cation Distribution for Phase Transformation

**Figure 2:**
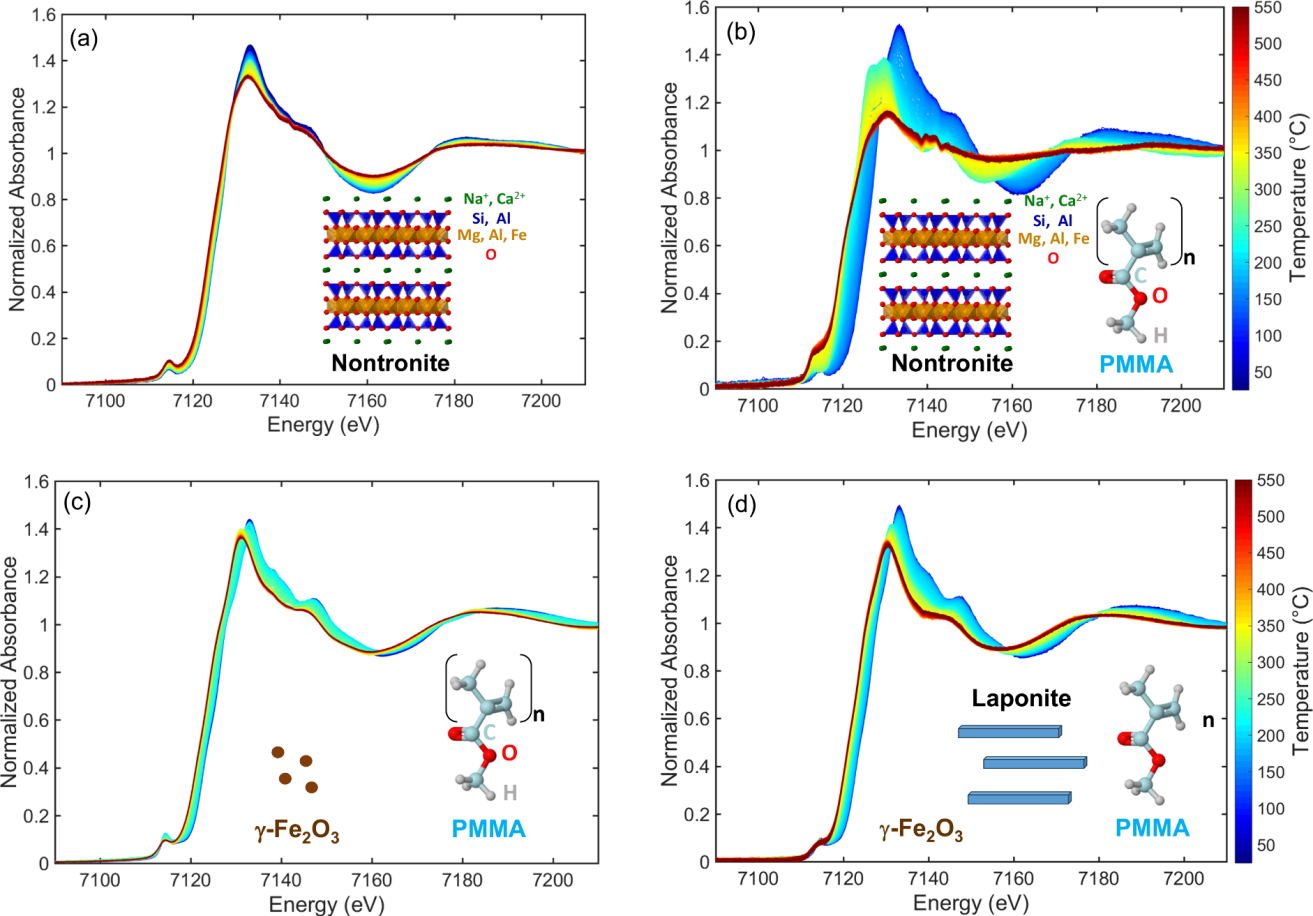
In situ Quick-XANES Fe K-edge monitoring of (a) pristine nontronite, (b) PMMA-Non, (c) PMMA-M, and (d) PMMA-M-Lap during heating from RT to 550 °C at 10 °C min^–1^. The color of the spectra shifts from blue at room temperature to red at 550 °C, as indicated by the temperature color bar scale. Schematic structural aspects of the phases are included as a guide for comparison.

To elucidate the relevance of the chemical distributions of iron atoms for thermal stabilization of the PMMA–clay samples, an in situ investigation using Quick-XANES and Raman spectroscopy was performed during heating of the materials from RT to 550 °C in a non-oxidizing atmosphere (N_2_). The XANES spectra for the pristine maghemite nanoparticles (Figure S1) remained invariant, while those for the pristine nontronite clay (Figure 2a) presented slight profile alterations, attributed to the release of iron from the octahedral sheets and the formation of a low crystallinity maghemite phase, as reported elsewhere.^47,48^ It is noteworthy that neither of the samples showed reduction of Fe(III) ions since the Fe K-edge rising edge position did not vary significantly when the maghemite and nontronite were heated. Therefore, all of the changes observed in the XANES spectra profiles during heating of the nanocomposites (Figure 2), especially the low-energy shift of the Fe K-edge rising edge, must have resulted from chemical reactions induced by the PMMA decomposition.

Significant differences in the evolution of the XANES spectra were observed, depending on the nature of the nanocomposite, with the more complex transformation of the Fe-bearing nontronite clay (PMMA-Non, Figure 2b) also affecting the XANES profiles, while a less significant change related to the iron atoms occurred for the PMMA-M and PMMA-M-Lap nanocomposites with embedded γ-Fe_2_O_3_ nanoparticles (panels c and d of Figure 2, respectively). In addition, the low-energy shift of the Fe K-edge rising edge absorption, an indication of iron reduction, was greater for the nanocomposites containing clays (Figure 2b,d), suggesting that the diffusion barrier effect provided by the clay exacerbated the reduction of iron.

**Figure 3:**
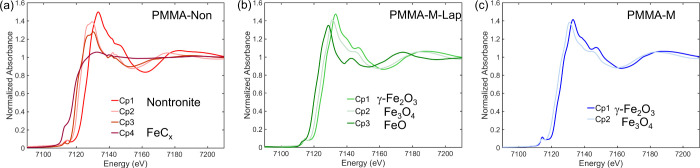
Pure XANES Fe K-edge spectra components, obtained by MCR-ALS minimization, during decomposition of the nanocomposites: (a) PMMA–nontronite, (b) PMMA–maghemite–laponite, and (c) PMMA–maghemite.

Multivariate MCR-ALS analysis of the data was applied to identify the iron species formed during thermal degradation of the PMMA nanocomposites between RT and 550 °C, using the XANES spectra and the changes in the concentration profiles with temperature. To determine the number of iron species involved in the decomposition during heating, the variance of the experimental data was first analyzed by PCA-SVD (Figure S2), which indicated the presence of four species for PMMA-Non, three species for PMMA-M-Lap, and two species for PMMA-M. Figure 3 compares the XANES spectra of the four, three, and two components isolated by MCR-ALS for PMMA-Non, PMMA-M-Lap, and PMMA-M, respectively. It could be concluded from this sequence of component numbers that the sparser the iron atom distribution in the fillers, the greater the number of intermediate iron phases resulting from reactions with the thermal decomposition products of the polymer matrix. The chemical nature of the different MCR components is discussed in more detail below.

### 3.3 Contribution of Iron Radical Trapping to Nanocomposite Thermal Stabilization

**Figure 4:**
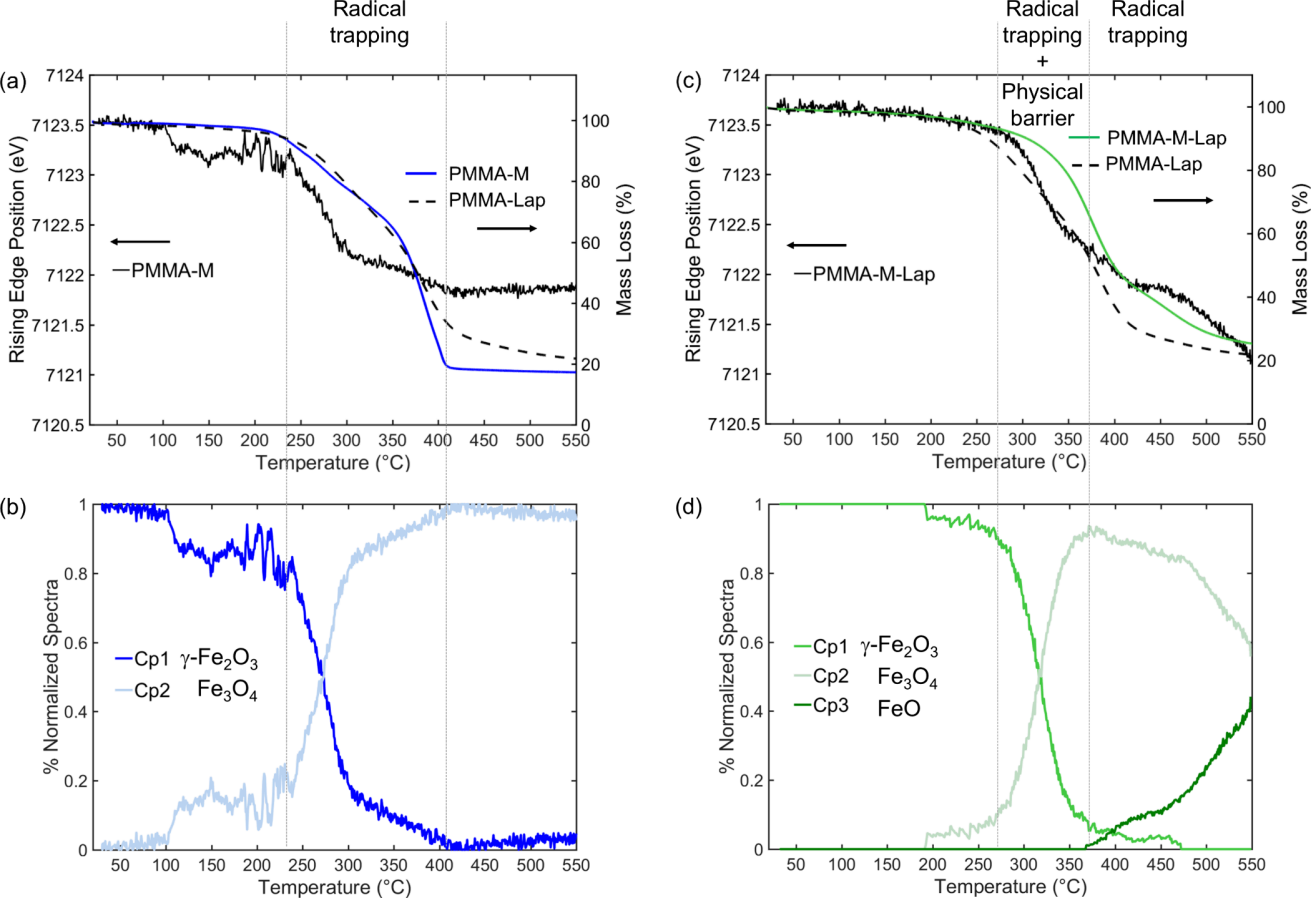
(a, c) Mass loss curves and energy positions of the Fe K-edge rising edge and (b, d) temperature dependence of the concentrations of the species derived from the XANES spectra for the PMMA-M and PMMA-M-Lap nanocomposites.

**Figure 5:**
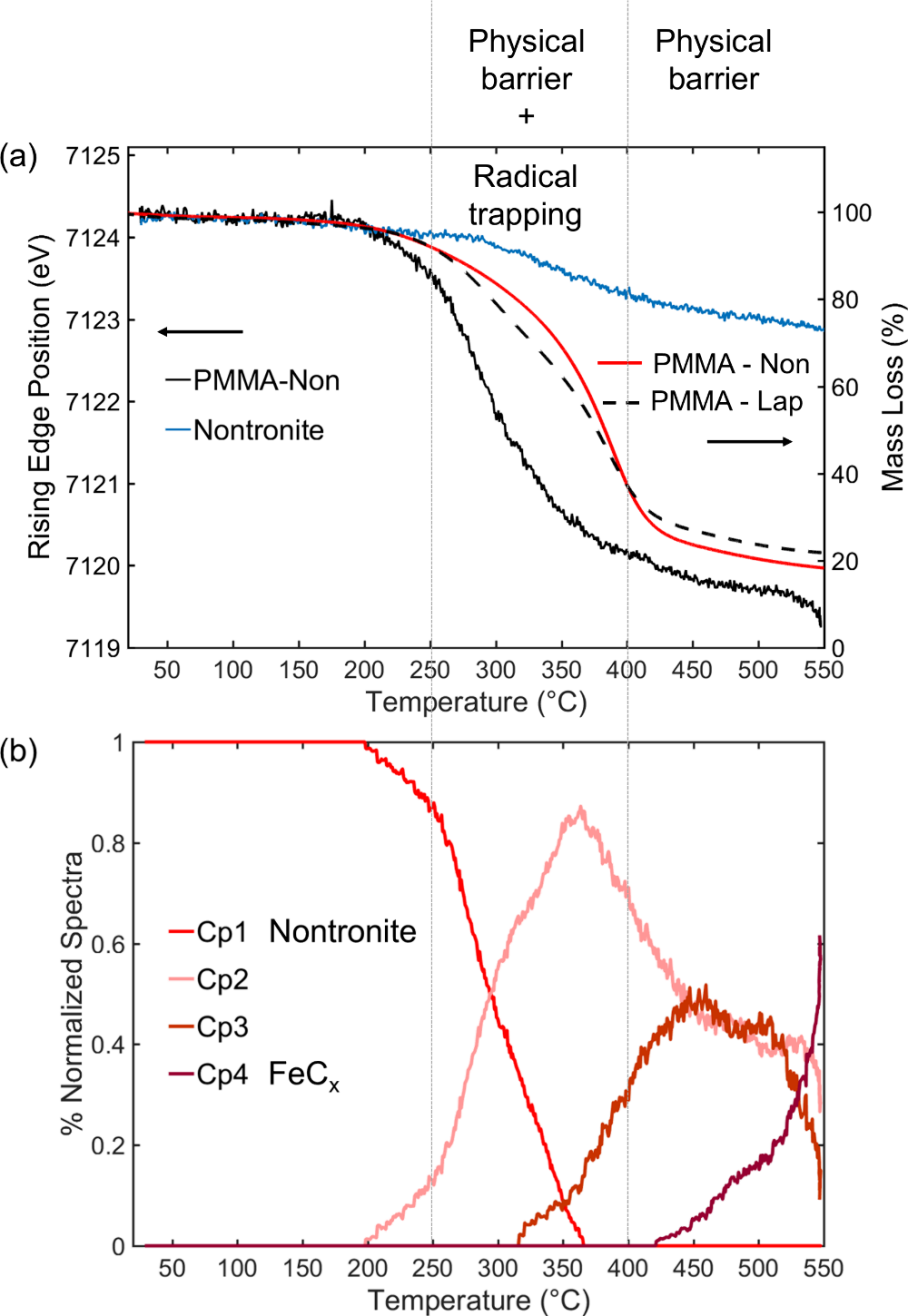
(a) Mass loss curves (PMMA-Non and PMMA-Lap) and Fe K-edge rising edge energy positions (PMMA-Non and nontronite). (b) Temperature dependence of the concentration of each species from MCR-ALS analysis of the XANES spectra. In (a), the Fe K-edge rising edge energy position for the nontronite clay (blue line) and the mass loss of the PMMA-Lap nanocomposite (dashed black line) are shown for the purpose of comparison.

The temperature dependences of the concentration profiles of the iron species, obtained from MCR-ALS analysis of the XANES spectra, are shown in Figures 4 and 5, together with the mass loss curves and the energy positions of the Fe K-edge rising edge for the different systems. Comparison of the energy position with the mass loss measured by TGA provided an indication of the contributions to thermal stabilization induced by radical trapping, for which a significant change of the iron oxidation state was observed, and by the physical barrier provided by the clay and/or the char formed by the degradation of PMMA. The PMMA-Lap nanocomposite, for which the mass loss result is shown for the purpose of comparison, can be considered an archetype of PCN, where the clay acted as a physical barrier to segmental motion of the polymer chains and to the mass transport of gases during the thermal degradation of PMMA.

Figure 4 shows the thermal stability coupled with the formation of iron species during the thermal degradation of the PMMA-M and PMMA-M-Lap nanocomposites. The reduction of maghemite is well documented in the literature^49^ and proceeds according to a stepped mechanism involving first the transformation of γ-Fe_2_O_3_ into spinel-like magnetite Fe_3_O_4_, followed by formation of wüstite FeO and finally metallic Fe. The Quick-XANES monitoring of the reduction of maghemite nanoparticles in 5% H_2_ (Figure S3) from RT to 550 °C enabled the isolation of the first and second intermediate oxide species mentioned above. The Cp2 components shown in Figure 3 for the PMMA-M and PMMA-M-Lap nanocomposites were identified as spinel-like magnetite Fe_3_O_4_, while the Cp3 component observed only for PMMA-M-Lap corresponded to FeO. Hence, the use of maghemite for preparing the nanocomposites did not apparently change the sequence of phase transformation of maghemite but affected the thermal stability of each phase. The spinel-like magnetite Fe_3_O_4_ formed by the reduction of γ-Fe_2_O_3_ in the PMMA-M nanocomposite was significantly more stable than that formed by thermal decomposition of the PMMA-M-Lap nanocomposite. The end of the mass loss for PMMA-M, at ∼405 °C, was coincident with the steady state for iron oxidation (Figure 4a).

For the PMMA-M-Lap nanocomposite (Figure 4c), the same invariance of the iron oxidation state was observed only from 405 to 475 °C, associated with a slower rate of nanocomposite mass loss. A further reduction was observed after 475 °C, with a more pronounced downshift of the Fe K-edge rising edge absorption at 550 °C for PMMA-M-Lap (−2.5 eV) compared to PMMA-M (−1.8 eV). This higher reduction of iron, attributed to the formation of wüstite FeO (Figure 4d), was due to more effective trapping of the radicals confined by the clay sheets, causing a significant stabilization of the PCN, with the mass loss only ending at 600 °C. The presence of laponite upshifted the temperature for the transformation of γ-Fe_2_O_3_ to Fe_3_O_4_ by about 60–70 °C (Figure 4). This evidenced the role of laponite in the recombination of radicals trapped by the clay, leading to a less reducing environment for iron species. However, it should be noted that above 410 °C, only the radical trapping by the iron accounted for the additional stabilization of the PMMA-M-Lap nanocomposite since the PMMA with laponite alone presented an almost invariant mass loss above this temperature. In summary, the greater thermal stabilization of the PMMA-M-Lap nanocomposite, compared to that of PMMA-Lap or PMMA-M, was strongly indicative of the synergic contributions of the layered clay edifice and the γ-Fe_2_O_3_ nanoparticles.

A larger shift in the energy of the Fe K-edge rising edge at 550 °C (about −4.5 eV) was observed for the iron species embedded in the PCN produced with Fe-bearing nontronite clay (Figure 5) compared to those prepared with maghemite (Figure 4). Carvalho et al.^29^ reported an edge shift of −3.5 eV for pristine nontronite subjected to heat treatment at 450 °C in a static air atmosphere, corresponding to Fe^3+^ → Fe^2+^ transformation. In the present case, with heat treatment at a higher temperature, the in-depth MCR-ALS analysis (Figure 3) revealed the formation of two Fe^2+^ intermediates with a rising edge shift of −4.2 eV, compared to the rising edge position for the Fe^3+^ species embedded in the as-prepared PCN, and an Fe^0^ intermediate, where the rising edge position was shifted by −7 eV from the initial position for the Fe^3+^ species, after 30 min of isothermal heating at 550 °C. Although there was significantly greater reduction of iron for the PMMA-Non nanocomposite during the degradation of PMMA, comparison of the TGA curves for PMMA-Non and PMMA-Lap (Figure 5) indicated that the gain in stability provided by the radical trapping (potentially linked to the reduction of iron) was relatively modest when compared to the physical barrier provided by the clay. Furthermore, the physical barrier contribution became dominant above 375 °C since the rate of reduction measured by the shift of the position of the absorption rising edge followed an evolution comparable to that observed for the pristine nontronite clay (Figure 5). A possible explanation for this poor radical trapping performance of iron was the occurrence of a concurrent reaction between iron and carbonaceous species derived from PMMA decomposition.

In the specific case of the PMMA-Non nanocomposite, Fe^3+^ in isovalent solid solution in the clay structure was stable up to 170 °C, above which the reduction of iron led to the progressive formation of three iron species, two containing Fe^2+^ that was partially transformed to Fe^0^ at 550 °C (Figure 5b). The formation of the first Fe^2+^ species occurred at the expense of Fe^3+^ present in the octahedral sheets of the clay. When almost all of the Fe^3+^ atoms had been consumed (at around 350 °C), the formation of a new Fe^2+^ species began from consumption of the first one. Concomitantly, the mass loss rate increased, suggesting that this transformation did not contribute to radical trapping. Above 420 °C, there was the start of the formation of metallic iron (black curve) from reduction of the second intermediate Fe^2+^ species, which coincided with a decreasing rate of PMMA mass loss.

### 3.4 Contribution of Carbonaceous Deposits to Nanocomposite Thermal Stabilization

**Figure 6:**
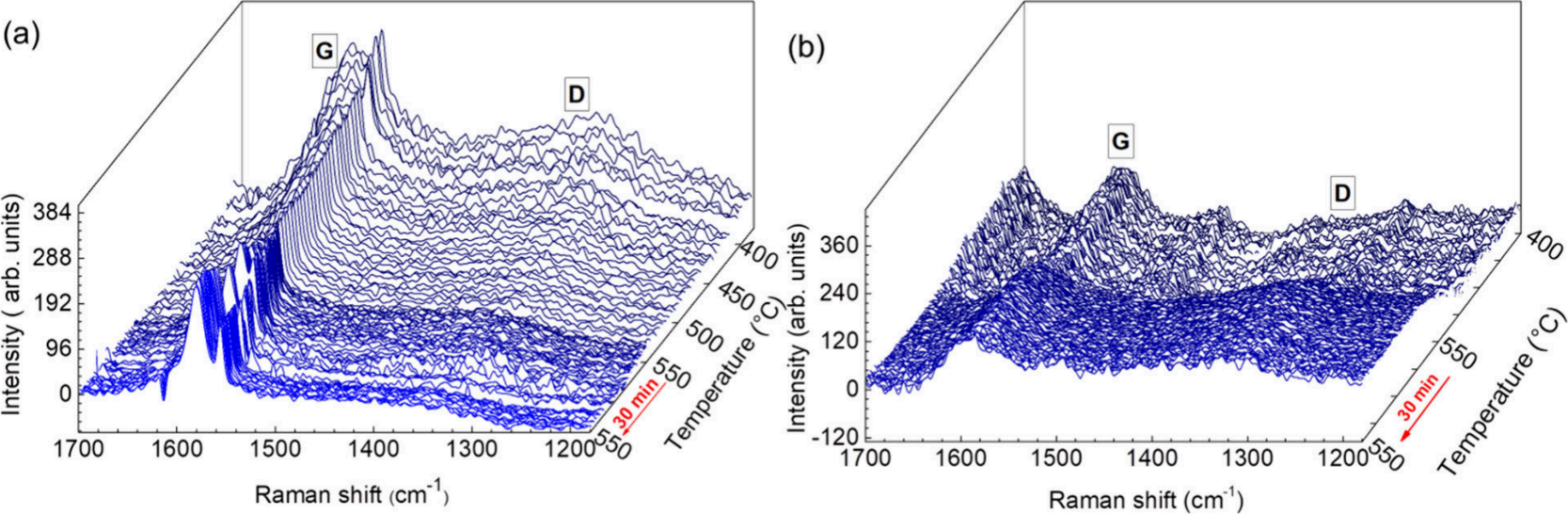
Raman spectra showing coke formation during decomposition of the PMMA-Non and PMMA-M-Lap nanocomposites.

The relevance of the possible reaction between iron and the carbonaceous species derived from PMMA decomposition was evaluated from the evolution of the Raman spectra (Figure 6) acquired simultaneously with Quick-EXAFS during the thermal decomposition of the PMMA-Non and PMMA-M-Lap nanocomposites. Unfortunately, it was not possible to monitor char formation for temperatures lower than 375 °C due to the strong fluorescence backgrounds of the samples rich in Fe^3+^. For PMMA-Non, high intensity characteristic D and G lines of char deposits were evident at ∼375 °C, while these lines were much less intense throughout the temperature range for PMMA-M-Lap. The G line located near 1590 cm^–1^ was attributed to graphitic-type carbon, while the D line located at around 1320 cm^–1^ was associated with in-plane graphitic imperfections.^50^ The dominant contribution of the G line observed below 375 °C for PMMA-Non was characteristic of ordered graphitic carbon, as in graphite layers, indicating the formation of graphitic coke deposits.^51^ Above 375 °C, the D lines were of low intensity and were similar for the PMMA-Non and PMMA-M-Lap nanocomposites. The presence of the G and D lines in the Raman spectra for the PMMA-Non sample was consistent with the formation of the iron species determined by EXAFS (Figure 5).

Therefore, it is proposed that the formation of almost 60% of iron carbide (cementite) resulted from the expulsion of iron from the nontronite sheets and its reaction with the carbonaceous species formed by PMMA decomposition. Additionally, the near-invariance of the G and D lines, as a function of temperature above 375 °C, indicated that the redox process involving the formation of metallic iron (Fe^2+^ → Fe^0^) occurred without the participation of graphitic coke present in the char. In other words, only the Fe^3+^ → Fe^2+^ reaction involving the release of iron atoms from the octahedral nontronite sheets actively contributed to the charring processes with the formation of the PMMA thermal decomposition products. This mechanism was consistent with the invariance of the G and D line profiles throughout the temperature range during the iron transformation observed for the PMMA-M-Lap nanocomposite.

Finally, the transformations observed for the PMMA-M and PMMA-M-Lap samples followed the iron oxidation–reduction diagram (Chaudron diagram), where γ-Fe_2_O_3_ → Fe_3_O_4_ → FeO. These diagrams delimit the thermodynamic stability boundaries of metal oxides at different temperatures as a function of the reducing potential of the gaseous atmosphere.^52^ It is interesting to note that wüstite (FeO) is metastable below 570 °C, while the addition of clay can increase its stability, providing protection against environmental disturbances and allowing for the occurrence of this phase of iron at low temperatures.^48,52^

## 4 Conclusions

The chemical reaction involved in radical trapping was clearly evidenced by a comparison of the TGA curve with the thermal evolution of the position of the Fe K-edge. The observed shift to lower energy of the absorption rising edge demonstrated that the extinction of polymeric radicals was due to transformation of the oxidation states of the iron cations in the clays, such as the reduction of Fe^3+^ to intermediates based on Fe^2+^ or to Fe^0^. The overlapping of the TGA and Fe K-edge rising edge energy curves at low temperatures indicated the existence of the clay lamellae barrier effect, while the tendency for overlapping at high temperatures was related to the additional physical barrier effect caused by the deposition of carbonaceous char.

The main mechanisms by which the smectite clay contributed to nanocomposite stabilization were the barrier effect and char formation, but in the absence of these, as in the case of the PMMA-M nanocomposite, the predominant mechanism was the trapping of radicals formed during PMMA decomposition. Despite the increased thermal stability induced by iron radical trapping, the association of the polymer and iron oxide nanoparticles, as for PMMA-M, was ineffective in preventing the formation of MMA during heating of the nanocomposite.

Reduction of the iron atoms present in the iron oxide nanoparticles was more efficient when assisted by the nanoconfinement of radicals by the lamellar clay nanostructure, as shown for the PMMA-M-Lap nanocomposite, where mass spectrometry analysis of the gas released during PMMA heating showed that the nanoconfinement provided by the lamellar structure of the clay prevented the release of the MMA monomer.

Finally, increasing the iron oxide dispersion by using smectite clay containing iron atoms in the octahedral sheets led to the greatest improvement in thermal stability, as observed for the PMMA-Non nanocomposite. This could be explained by the combined effects of the four contributions, namely the diffusion barrier provided by the clay lamellae, the radical trapping by iron, the additional barrier created by the deposition of graphitic char, and the abstraction of carbon from the char by the iron carburization reaction, which hindered CO_2_ formation by oxidative coking. The combined action of these mechanisms should contribute to reducing the speed of fire propagation and the release of toxic gases, increasing the practical efficiency of evacuation and rescue in emergency situations.

## Supporting Information Available

ICP-OES elemental analysis of metals present in the clay and oxide fillers; monitoring of the maghemite phase by Quick-XANES at the Fe K-edge during heating; PCA scree plots for PMMA-Non, PMMA-M-Lap, and PMMA-M; and XAS spectra of γ-Fe_2_O_3_, magnetite Fe_3_O_4_, and wüstite FeO (PDF)
