# Supplementary material for: Relevance of the Iron Distribution in Natural Smectite Clays for the Thermal Stability of PMMA–Clay Nanocomposites
Source: ACS Omega. 2024 Aug 16;9(34):36579–88. doi: 10.1021/acsomega.4c04751 (PMC11360043; doi:10.1021/acsomega.4c04751)
Supplement: Supplementary file 1 — ao4c04751_si_001.pdf [file ao4c04751_si_001.pdf]

Supporting Information

Relevance of the iron distribution in natural smectite clays for the thermal stability of PMMA-clay nanocomposites

Camila R. Ferreira[1,2], Celso V. Santilli[1], Valérie Briois[2], Sandra H. Pulcinelli[1]*

[1]*Universidade Estadual Paulista, Instituto de Química de Araraquara, SP, Brazil*

[2]*Synchrotron SOLEIL, UR1-CNRS-SOLEIL, L'Orme des Merisiers, Saint-Aubin, France*

E-mail addresses: sandra.h.pulcinelli@unesp.br

camila.raiane@unesp.br

Table S1. ICP-OES elemental analysis of metals composing the clay and oxide fillers (in wt.%), content of nanofillers for nanocomposite preparation, and sample nomenclature.

Nanofillers	Li	Na	Mg	Al	Ca	Fe	Nanofillers (%)	Nomenclature
Nontronite	-	0.01	9.00	0.31	0.32	5.58	15	PMMA-Non
Laponite	3.30	1.91	14.30	-	0.15	-	15	PMMA-Lap
Maghemite + laponite	2.56	1.48	10.90	-	0.15	8.47	15	PMMA-M-Lap
Maghemite	-	-	-	-	-	-	15	PMMA-M

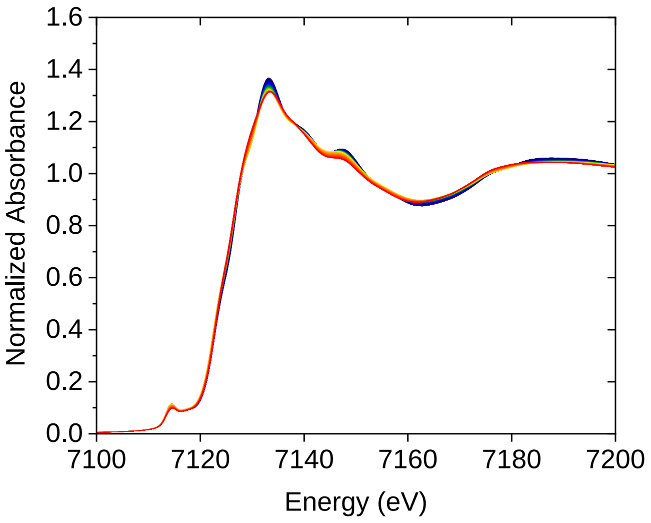

Fig. S1. Monitoring by Quick-XANES at the Fe K-edge during heating of the maghemite phase between RT and 550 °C at 10 °C min^{-1}.

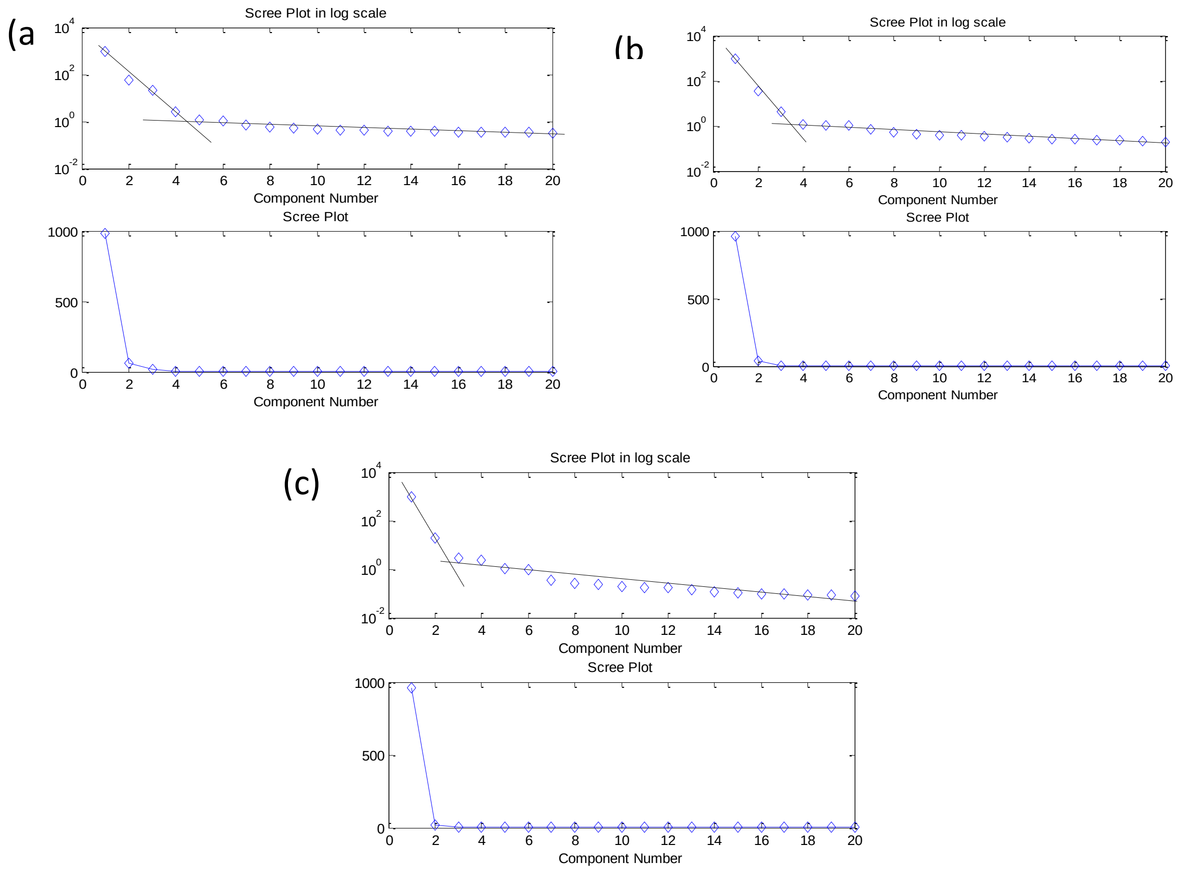

Fig. S2. PCA scree plots for the (a) PMMA-Non, (b) PMMA-M-Lap, and (c) PMMA-M nanocomposites decomposed in an N_2 atmosphere.

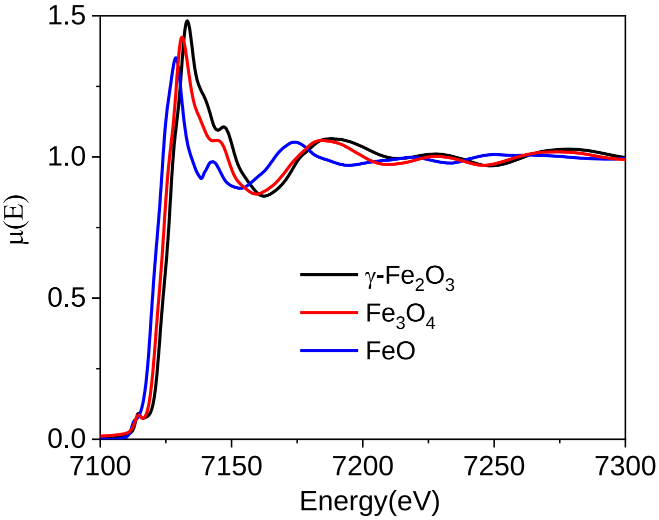

Fig. S3. XAS spectra of γ-Fe_2O_3, spinel-like magnetite Fe_3O_4, and wüstite FeO, extracted from MCR-ALS multivariate analysis of the data set acquired during heating of maghemite nanoparticles from RT to 550 °C, in an atmosphere with 5% H_2.
